# Formulation and acceptability of local nutrient‐dense foods for young children: A formative study for the Child Health, Agriculture and Integrated Nutrition (CHAIN) Trial in rural Zimbabwe

**DOI:** 10.1111/mcn.13605

**Published:** 2023-12-13

**Authors:** Dexter T. Chagwena, Shamiso Fernando, Naume V. Tavengwa, Shadreck Sithole, Chandiwana Nyachowe, Handrea Njovo, Kavita Datta, Tim Brown, Jean H. Humphrey, Andrew J. Prendergast, Laura E. Smith

**Affiliations:** ^1^ Zvitambo Institute for Maternal and Child Health Research Harare Zimbabwe; ^2^ Ministry of Health and Child Care Harare Zimbabwe; ^3^ School of Geography Queen Mary University of London London UK; ^4^ Johns Hopkins Bloomberg School of Public Health Baltimore Maryland USA; ^5^ Department of Public and Ecosystem Health Cornell University Ithaca New York USA

**Keywords:** child nutrition, complementary feeding, infant feeding behaviour, low income countries, nutrition education, qualitative methods

## Abstract

Stunting affects almost one‐quarter of children globally, leading to reduced human capacity and increased long‐term risk of chronic disease. Despite intensive infant and young child feeding (IYCF) interventions, many children do not meet their requirements for essential nutrients. This study aimed to assess the feasibility of implementing an IYCF intervention utilizing nutrient‐dense powders from egg, biofortified sugar beans and *Moringa oleifera* leaf in rural Zimbabwe. A mixed‐methods formative study was conducted comprising the following: (i) a recipe formulation trial, (ii) trials of improved practices to assess acceptability of the intervention, and (iii) a participatory message formulation process to develop counselling modules for the IYCF‐plus intervention. Twenty‐seven mother–baby pairs were recruited between November 2019 and April 2020. Key domains affecting IYCF practices that emerged were time, emotional and physical space, cultural and religious beliefs, indigenous knowledge systems and gender dynamics. Household observations and sensory evaluation indicated high acceptability of the new ingredients. Recipe formulation and participatory message formulation by participants instilled community ownership and served to demystify existing misconceptions about the new food products. Families noted the potential for intervention sustainability because the foods could be grown locally. Supplementing complementary foods with nutrient‐dense local food ingredients as powders has the potential to sustainably address nutrient‐gaps in the diets of young children living in rural lower‐ and middle‐income countries. Comprehensive IYCF counselling utilizing a gender‐lens approach, family support and indigenous knowledge systems or resources are key elements to support positive behaviour change in complementary feeding interventions.

## Key messages

- Incorporating locally available solid foods into complementary feeding is a major challenge for caregivers in rural communities. There are several barriers that hinder mothers from feeding nutrient‐dense solid foods such as animal source foods, fruits and vegetables, resulting in nondiversified complementary diets.
- Formulation of nutrient‐dense, high‐quality protein complementary meals using powdered locally available food supplements is feasible, well tolerated and allows young children to be fed nutrient‐dense complementary meals in rural LMICs.
- Utilizing traditionally recognized functional foods such as *Moringa olifeira* encourages the involvement of elderly women/grandmothers in infant and young child feeding, enabling support for mothers.
- Integrated complementary feeding interventions incorporating behaviour change communication, provision/promotion of specific nutrient‐dense complementary foods (SQ‐LNS and local foods as powdered supplements), family support and agricultural production has potential for success in rural settings.

## Graphical abstract

This study describes the formulation, acceptability and feasibility of an IYCF‐plus intervention utilizing powders from nutrient‐dense local foods, SQ‐LNS for young children and behaviour change counselling.

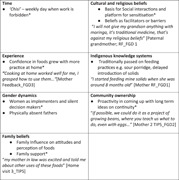

## 1 INTRODUCTION

Stunting is the most common form of undernutrition globally, affecting over 149 million children under the age of 5 years. Stunting is associated with increased child mortality (Black et al., 2013; Victora et al., 2021), reduced human capital and increased long‐term risk of chronic disease (Black et al., 2008; World Health Organization, 2019). Zimbabwe faces a multiple burden of undernutrition: over a quarter of children under 5 years are stunted (Zimbabwe National Statistics Agency [ZIMSTAT] & United Nations Children's Fund [UNICEF], 2019) and deficiencies of micronutrients such as iron affect seven out of 10 under‐five children (MoHCC, 2015). Stunting peaks during the period of complementary feeding from 6 to 24 months of age (Prendergast & Humphrey, 2014), driven partly by poor diets, which lack micronutrients and high‐quality protein (animal‐source foods [ASFs]), and poor feeding practices employed by caregivers (Chagwena et al., 2020; Speedy, 2003).

Suboptimal infant and young child feeding (IYCF) practices in low‐ and middle‐income countries (LMICs) limit the potential to curb stunting, child morbidity and mortality (Black et al., 2013; Heidkamp, 2017; Victora et al., 2021; White et al., 2017). The most common IYCF gaps include feeding nondiversified and low‐nutrient diets, especially those lacking essential food groups such as animal‐source, iron‐rich and vitamin A‐rich foods (Desai et al., 2015). Several IYCF interventions have been implemented with modest impact on linear growth (Adu‐Afarwuah et al., 2007; Dewey, 2013; Keats et al., 2021; Shekar et al., 2021). An intensive IYCF intervention in rural Zimbabwe as part of the Sanitation Hygiene Infant Nutrition Efficacy (SHINE) trial (Humphrey et al., 2015), which comprised community health worker (CHW)‐delivered behaviour‐change communication plus daily small‐quantity lipid‐based nutrient supplements (SQ‐LNS), reduced stunting by 20% (Humphrey et al., 2019). A higher proportion of mothers receiving the IYCF intervention fed their children diets that met minimum dietary diversity, increased ASFs and foods rich in iron and vitamin A (Humphrey et al., 2019). Intake of micro‐ and macronutrients was significantly higher among children in the IYCF intervention groups compared with non‐IYCF groups when SQ‐LNS was included, but when SQ‐LNS was excluded from the analysis, there was no difference in nutrient intake, indicating there was no additional benefit of the behaviour change component (Fundira et al., 2019). Thus, despite intensive promotion of messages addressing cultural barriers to complementary feeding, emphasizing that young children should consume diverse diets, can be fed any food that adults eat, and can be fed locally available foods (Desai et al., 2015; Dewey & Adu‐Afarwuah, 2008; Paul et al., 2012) children's diets were deficient in micronutrients. Overall, 32%, 73% and 23% of children did not meet the energy, folate and iron requirements, respectively (Fundira et al., 2019).

New IYCF interventions are needed that fill key nutrient gaps, to improve child growth and development. In particular, solutions based on locally available foods for young children are needed, and especially in the sub‐Saharan Africa region. (Lassi et al., 2020; Lutter et al., 2013). Cultural barriers and poor maternal capabilities including feeding skills, self‐efficacy and lack of family support hinder optimal complementary feeding (Matare et al., 2021; Paul et al., 2012). This formative work lays the foundation for the Child Health, Agriculture and Integrated Nutrition (CHAIN) trial, which is testing the impact of SQ‐LNS together with a package of locally available food powders and IYCF counseling (‘IYCF‐Plus’) designed to close nutrient gaps among young children in rural Zimbabwe. The CHAIN trial utilizes nutrient‐dense powders made from egg, biofortified sugar beans and *Moringa oleifera* leaf mixed with locally available foods such as mashed potatoes, thick consistency porridge and soup. Powdered food supplements increase the nutrient density of complementary foods, can be stored for long periods without refrigeration and allow individual packaging facilitating effective use in situations of nonoptimal hygiene conditions (Affonfere et al., 2021; Dewey & Brown, 2003; Dibari et al., 2012). Furthermore, nutrient‐dense powdered complementary foods allow for meeting children's nutrient needs by feeding small quantities of food during this period when the stomach capacity is small. In the study area, most complementary foods tend to be bulky, mainly cereal‐based, with low nutrient density. Hence, powdered supplements made it feasible to feed nutrient‐dense foods without increasing the amount of food consumed by the child (Nestel et al., 2003; Okoth et al., 2017; Owino et al., 2008). Before the implementation of CHAIN, the feasibility and acceptability of using these local, nutrient‐dense food powder supplements among mothers and children in a rural LMIC community needed to be tested. In this paper, we present the results of this formative study.

## 2 METHODS

### 2.1 Study site, participants

We conducted a mixed methods formative study in the district of Shurugwi in Zimbabwe between October 2019 and March 2020. This is a predominantly rural subsistence farming and small‐scale mining area located in Midlands Province, with a rural population of 77,570 people (ZIMSTAT, 2012) and high food insecurity (30%) (IPC, 2020). The goal of the study was to assess the acceptability and feasibility of delivering locally available foods as powdered supplements, to close the nutrient gap in diets fed to rural Zimbabwean children. To achieve this, we conducted three consecutive qualitative studies: (i) a recipe formulation (RF) trial, (ii) trials of improved practices (Dickin et al., 1997) and (iii) participatory IYCF message formulation.

### 2.2 Study rationale

We conducted this formative study to inform the CHAIN trial. The design and rationale of the trial has been described elsewhere (Smith et al., 2022). In brief, CHAIN is an unblinded individually randomized controlled trial among 192 households in the same district, which selects children aged 5–6 months to receive IYCF or ‘IYCF‐plus’ between infant ages 6 and 12 months. IYCF comprises ground white maize‐meal and SQ‐LNS together with education on complementary feeding. IYCF‐plus comprised pro‐vitamin A maize‐meal and SQ‐LNS, plus moringa olifeira leaf powder, whole egg powder and biofortified sugar bean powder. All interventions were delivered by CHWs during home visits to children up to 12 months of age. The trial will report outcomes in 2023.

### 2.3 Formative study design

**Figure 1:**
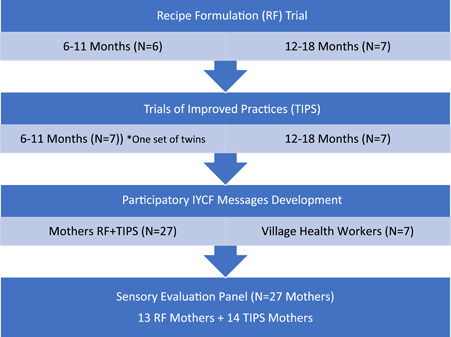
Flow chart of formative study participants.

The flow of study procedures is shown in Figure 1. Three qualitative studies were conducted in sequence at a central community clinic and were led by a nutritionist and a social science researcher. In total, 27 mothers with children between 6 and 18 months of age were identified by seven CHWs working near the community clinic. Each CHW selected at least three mother‐child pairs to participate in the study employing random selection from their catchment area. To be eligible for the study, infants also needed to be free from disabilities that interfered with feeding. Mothers were enrolled by CHWs and provided written informed consent. Two focus group discussions (FGDs) were conducted before and after each trial. The initial discussion focused on current IYCF practices and perceptions of the proposed new food ingredients, and the follow‐up discussion captured feedback from mothers after exposure to the new complementary feeding recipes and interventions. All interview sessions were audio recorded.

#### 2.3.1 RF trial

RF and standardization (Pan American Health Organization & UNICEF, 2013) was conducted with 13 mother‐child pairs. Mothers were divided into two groups: mothers of children aged 6–11 months (Group 1; *N* = 6), and mothers of children aged 12–18 months (Group 2; *N* = 7). The goal of the RF trial was to produce at least two ways of preparing a meal with each of the three new foods that was acceptable to mothers and infants. These were recipes suitable for the younger children (6–11 months) and the older children (12–18 months). Six recipes (four porridge, one mashed potato, one thick consistency soup) from the new food ingredients were formulated and standardized. The porridge and soup were of thick consistency, appropriate for infant and child feeding.

#### 2.3.2 Trial of improved practices (TIPs)

Two groups of 14 mothers of children aged 6–11 months (*N* = 7) and 12–18 months (*N* = 7) participated in TIPs. These were separate mother–baby pairs from the RF trial. The TIPs approach is used to evaluate acceptance and adoption of newly promoted behaviours utilizing a small number of people before promoting the behaviour widely at the community or population level (Dickin et al., 1997). The TIPs intervention included a cooking demonstration on the new recipes and a 7‐day period to try out the new complementary feeding recipes. Preparation of each of the six new recipes was demonstrated to both groups of mothers. Following cooking demonstrations, mothers prepared one or more of the new recipes (individually) and fed their child. The quantity of the food prepared, consumed, and left over by the child, was measured using a food scale and recorded. Reactions from mothers and their children to the food recipes were discussed as a group and recorded by researchers. In cases where a child rejected the new food, the recipe was modified and offered to the child again on the same day (e.g., if a child refused porridge with moringa, the mother would prepare the recipe again, with less moringa powder and offer it to the child again).

Mothers were provided with adequate supplementary food powders to take home and try out the new recipes over 7 days. During this period, the researchers and CHWs conducted four home visits to each mother to observe how preparation and feeding were employed. One of the home visits was a combined visit from the two researchers and a CHW. During home visits, researchers observed and recorded how mothers stored the food powders, prepared the meals, and fed their children. Interactions with other family members were observed and recorded. The researchers also conducted discussions with family members to understand their perceptions and feelings regarding the new recipes. During the home visits, CHWs assisted mothers with preparation of food recipes, discussed how the child was tolerating the new foods, and gathered feedback from family members. Problem recipes were modified following recommendations from mothers, for instance mothers realized it was better to use cold water to mix bean and moringa powder and lukewarm water to mix egg powder to improve smoothness of the porridge for young infants being introduced to solid foods at 6 months.

#### 2.3.3 Participatory IYCF message development

A participatory message development approach was employed to generate IYCF messages to be included in intervention modules for the CHAIN trial. The messages were developed with the aim of providing awareness and knowledge of the food supplements for the CHAIN trial. All 27 mothers who participated in the RF and TIPS trials were split into three groups to develop messages on provitamin A maize (not provided but available in the community), egg powder, bean powder and moringa leaf powder. Mothers formulated messages supported with images they thought would be effective to communicate to caregivers regarding the new food ingredients, how to prepare the new recipes and ways to feed young children at home. Data from the RF and TIPs FGDs were used to identify knowledge gaps, negative attitudes and barriers that could influence uptake of new foods. Using example messages and images from the UNICEF and SHINE trial IYCF counselling packages, mothers formulated relevant messages, described and provided sketches of appropriate images to go together with these messages to address knowledge gaps, negative attitudes and perceptions exhibited towards the new food recipes and ingredients. A similar participatory co‐creation process of IYCF counselling tools and materials was conducted with the same group of seven CHWs. The messages were then formulated into IYCF counselling modules through a participatory process involving district‐level and community‐level health workers. These health workers included the district nutritionist and health promotion officer, community nurses, CHW trainers and a social worker. The messages were compiled into an IYCF counselling module booklet for use in the CHAIN trial.

#### 2.3.4 Sensory evaluation

Sensory evaluation was conducted following the formulation and standardization of recipes. All 27 women who had participated in the RF and TIPS qualitative studies made up the sensory evaluation panel. The women evaluated the food recipes for properties of appearance, smell, taste, and consistency, each using a five‐point written Hedonic scale (from ‘5’—*like very much*, to ‘1’—*dislike very much*) (Lim, 2011).

#### 2.3.5 Data analysis

All FGDs and household interviews were audio recorded, transcribed, and translated from Shona into English (full verbatim). Transcriptions were converted into MS word and loaded on NVivo (version 12.0). Thematic content analysis was utilized to synthesize the data. Data were first coded, grouping similar ideas together into themes. The codes were created through identification of key issues from the interviews (Neuendorf, 2018). Coding was conducted by two researchers, one coding the data and the second researcher verifying the codes. Quantitative data on sensory evaluation and quantities of foods consumed were processed using Microsoft Excel.

#### 2.3.6 Ethics

This formative research study was approved by the Medical Research Council of Zimbabwe (MRCZ/A/1675). Dissemination of trial results will be conducted through the Community Engagement Advisory Board in the study district and through national‐level platforms.

## 3 FINDINGS

The 27 caregivers (26 mothers and one grandmother) who participated in the study were aged between 17 and 43 years (Figure 1). All were married and one‐third (*N* = 9) were living with extended family members. Twenty (74%) were first‐time mothers. Almost three‐quarters (*N* = 20) of mothers reported living alone for long periods, as they were married to small‐scale or artisanal miners. A total of six focus group discussions were conducted before and after each qualitative trial to explore initial maternal perceptions of the proposed complementary foods and practices, and after the trial to assess feedback. Analysis of the interview transcripts revealed four main themes related to participants' views on the introduction of new foods to their children's diets:(1) acceptability and perceived advantages of using biofortified bean, egg and moringa powders as additional foods for young children; (2) role of men and the extended family; (3) influence of indigenous knowledge systems; and (4) knowledge gaps on IYCF.

### 3.1 Theme 1: Acceptability and perceived advantages of using bean, egg and moringa powder as additional foods for young children

Most study participants expressed positive attitudes towards the use of bean, egg and moringa powders in preparing food for their young children.

‘I would love to see how it comes out…I am not sure if my little one will like this green colour and the smell’ [Mother 7; RF, FGD1].

Almost all caregivers (*N* = 26) were eager to try the new foods. Most caregivers reported that taking the ingredients to try out at home helped gain confidence in them. The most often reported confidence booster was witnessing the child's positive response at first taste of the porridge with all three ingredients.

‘My son does not really like porridge at home, but look at how he cleared his plate, can I have some more of this one’ [Mother 11, TIPS, FGD2]

Some caregivers reported sharing the food powders with neighbours because of the perceived benefits of adding these powders to the children's food. Sharing also facilitated uptake of the new foods based on the positive feedback from neighbours and extended family. An additional indicator of uptake included the pro‐active comments from participants regarding their desire to grow and process the foods into powders for their children and the rest of the community.

‘Maybe we can farm in groups according to where we live; you can teach us how to grind the crops into powder form’ [Mother 10; RF, FGD1]

Most participants indicated time as an enabling factor to acceptance of the new food ingredients. Participants reported that it took no extra time for preparation of complementary meals after incorporating the food powders and utilizing a similar cooking routine.

‘I will not give my grandson anything with moringa, it's traditional medicine, that's against my religious beliefs’ [Caregiver 3; TIPS, FGD2].

Caregivers expressed religious beliefs as a major barrier to acceptance of the new food supplements. This caregiver initially only agreed to try the other powders except moringa, but later changed her mind after participating in the message development exercise where she was part of the group that described the process of how moringa powder was made.

#### 3.1.1 Sensory characteristics of food recipes

**Figure 2:**
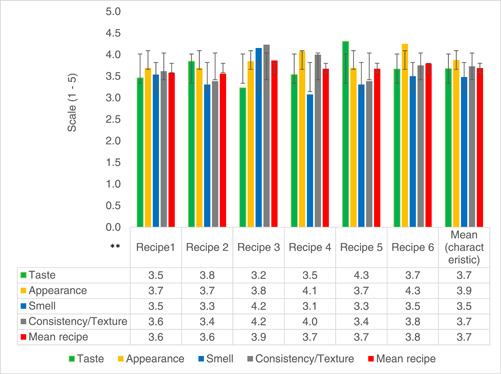
Sensory characteristics of recipes evaluated by mothers who had limited exposure participating in the Recipe Formulation (RF) Group. *Limited exposure was classified based on the limited time mothers were exposed to the new food recipes while participating in the RF study. They prepared the food recipes at the central clinic point during RF and were not provided food supplements to take home before the sensory evaluation exercise. **Recipe 1 to 4 = thick consistency porridge. Recipe 5 = thick consistency soup. Recipe 6 = mashed potatoes with powders.

All six formulated and standardized recipes were found highly acceptable by the sensory evaluation panel. Taste, smell, appearance, and consistency were rated at least 3.5 out of 5 by mothers (Figure 2).

**Figure 3:**
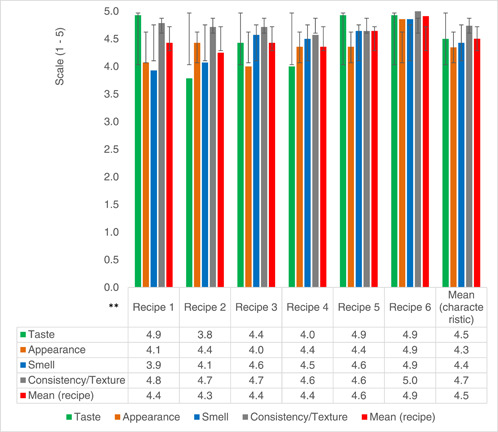
Sensory characteristics of recipes evaluated by mothers with enhanced exposure participating in the Trials of Improved Practices Group. *Enhanced exposure was classified based on the increased time mothers were exposed to the new food recipes while participating in the TIPS study (they prepared the food recipes at home for 7 days). **(Recipes 1–4 = thick consistency porridge. Recipe 5 = thick consistency soup. Recipe 6 = mashed potatoes with powders).

Acceptability by mothers in the TIPS study, who took supplements home for 7 days and therefore had more exposure to the food recipes, was higher (range 4.4–4.9 on a 5‐point hedonic scale) (Figure 3). These mothers had the opportunity to get used to preparing these food supplements, knowing exactly when to add powders, and ideal cooking times. Concerns on acceptability of green‐coloured porridge in the community were noted. There was a perceived fear that young children could vomit or have stomach problems after consuming moringa leaf powder, although no adverse effects were observed (Table 1). However, excitement by caregivers on feeding green porridge with moringa overcame these fears. One mother said:

‘This green porridge is colourful and tasty cause of moringa, that it has made it easier for my child to like to eat porridge. He never used to like porridge this much’ [Caregiver; TIPs, FGD2].

**Table 1:** Key knowledge and practices gaps in IYCF.

**Knowledge gaps on IYCF**
Lack of knowledge and awareness on biofortified or vitamin–mineral‐rich complementary foods.
Mothers lacked knowledge of the concept of grinding nutrient‐rich foods for young children, for example, dried kapenta, mopane worms and so on.
Importance of thick porridge was not commonly known among mothers. Mothers prepared thin porridge.
Mothers lacked ideas and initiatives to prepare nutrient‐dense complementary meals.
IYCF was not observed as an important task that families had to plan or were prepared for. Gender‐sensitive approaches could be incorporated addressing involvement of family members and fathers/male family members. Gendered messages and support were designed as part of CHAIN IYCF modules.
**IYCF practice gaps**
Mothers did not feed solid protein‐rich foods between 6 and 11 months, such as meat, eggs and beans. They were perceived as foods for older children who could chew. Instead, mothers fed young children thin soup made from these foods. Eggs were not usually fed to young children due to high cost, incorrect advice from some health workers and cultural beliefs prohibiting feeding of eggs to children.
Feeding thin or runny porridge by mothers.
Early introduction of solid foods before 6 months. Most CHWs and mothers reported that exclusive breastfeeding was not commonly practiced. This practice gap was not addressed in the CHAIN trial BCC modules as children are recruited at 5 months regardless of Exclusive Breastfeeding status.
Early weaning before 24 months was reported as common.
**Economic‐related gaps**
Lack of financial resources to purchase the recommended nutrient‐dense foods or prepare them into powders.
Absence of ready markets to sell agricultural products meaning less income or earnings to purchase the complementary food and proposed powders.
**Knowledge and practice gaps on new food ingredients (bean, egg and moringa leaf powder) likely to affect the CHAIN trial**
Sharing was common among household members—child siblings, elders and neighbours. The need for provision of a sharing ration and emphasis on feeding adequate rations for the child was observed.
Fear or reluctance among some mothers/caregivers to try new foods, for example, moringa leaf powder and egg powder.
Moringa leaf powder was regarded as a spice. This could likely result in reduced moringa powder fed to young children.
Reluctance by apostolic sect families to use moringa leaf powder as the tree was regarded as a herb. Use of herbs was discouraged in their religion.
Concerns on acceptance of green coloured porridge in the community used to brown or white porridge was noted. There was fear that young children could vomit or have stomach problems after consuming moringa leaf powder.
Early cessation of moringa by mothers of young children 6–9 months as some younger children passed on green coloured stool in the initial days of commencing foods with moringa leaf powder.
Concerns on feeding moringa to HIV‐infected children was raised by mothers.

Abbreviations: CHAIN, Child Health, Agriculture and Integrated Nutrition; HIV, human immunodeficiency virus; IYCF, infant and young child feeding.

Acceptance of the new complementary foods was high among infants and young children. Children enjoyed the thick porridge, thick soup and mashed potatoes, consuming larger amounts of these dishes than the foods they usually ate at home. Mothers reported that moringa improved children's appetite and increased food consumption. The average amount of porridge consumed by children during each meal was 135 g (SD 19) in the younger group (6–11 months) and 533 g (SD 65) in the older group (12–18 months).

### 3.2 Theme 2: Role of men and the extended family

Participants observed at home were open to discuss the introduction of new foods. Some mothers were confident to use moringa in their children's diets following positive assurance and encouragement from in‐laws and partners. Other family members were involved during the introduction of new supplement powders, especially moringa powder, due to the interest in these functional foods. Moringa trees were present among some households' gardens and moringa was known to improve health, but many were not aware of potential benefits in complementary feeding. When asked, most fathers thought it could benefit young children. It was also believed to be an aphrodisiac attracting interest of many.

Men in this area largely participate in small‐scale and informal artisanal mining, which entails prolonged physical absence of fathers and decision‐makers within households. This granted women the position of implementers, creating a unique culture within this community. This contests the assumption that in rural areas within LMICs, men possess overall decision making on child feeding, as they may have an artificial sense of control or an assumed silent role.

‘My husband just has to make sure we have everything we need; I give him a list and he buys, but I decide which meals I want to prepare’ [Mother 3; TIPS, FGD2]

‘It was easy for me try out these new powders because my husband does not interfere in child feeding. He lets me make the decisions and only makes sure we have the money to buy food. Of course, another important factor was that these new recipes did not require more time to prepare, so the need for resources such as cooking gas, firewood or water did not change, making it easier to accept them. It is my husband's duty that we have enough firewood and water at the house for me to prepare food easily’ [Mother; TIPS, FGD2].

Caregivers who lived without extended family had more autonomy than those who shared space with extended family. They were able to learn more about the new foods and follow the recipes in the manner they were taught. Caregivers who lived with extended family reported that at times, family members imposed their ways of cooking or insisted on using the food powders for other purposes.

‘I am blessed that I do not live with my mother‐in‐law and my husband is usually away from home since he is into mining. That way I have enough freedom to decide on how I should care for my baby or what I can feed my baby. So, when I was given these powders I could easily try them out at my own pace without any influence because I am the one who knows best what benefits my child’ [Mother; TIPS, FGD2].

### 3.3 Theme 3: Influence of indigenous knowledge systems on adoption of promoted complementary feeding practices

Child‐feeding practices are based on cultural beliefs passed‐on in the community and across generations within families. For instance, some caregivers reported delaying introducing solid foods until a child was at least 7 months of age. Even upon delayed introduction of solids, children would only be fed thin maize‐meal porridge with no other food additives. Mothers believed this was the appropriate food for infants and only introduced other foods such as eggs and beans towards the age of 12 months. Foods such as beans, meats and eggs were commonly available in their communities and consumed, but not regarded as appropriate complementary foods. Also, fruits and vegetables were rarely fed to young children, as mothers believed that young children could not chew and swallow solid foods. Mothers reported that avoiding feeding fruits and vegetables had always been their practice and had been passed on from old generations. Besides some cultural beliefs hindering feeding of eggs to young children, eggs were considered expensive and most families could not afford them.

Grandmothers showed interest in using moringa as complementary foods and this excitement was observed among other family members as well. Most elderly women thought that as traditional foods, including moringa, were now being promoted by health workers, this would result in grandmothers becoming more involved in child feeding and supporting mothers (without any suspicions previously observed between mothers and grandmothers).

One grandmother said:

‘You see, now even your nurses and clinics are now recognizing our traditional foods such as moringa. They have now come around to realize our old ways are helpful to the health of children. It means we will work well together [with our daughters and daughters‐in‐law] to look after our grandchildren’ [Grandmother; TIPs, Home Visit]

The idea that modern health practitioners were realizing the role of indigenous resources in child nutrition was satisfying to most elderly people. This created a sense of trust and a certain level of approval for mothers to practise freely what they were taught from the health facilities without being questioned by grandmothers or mothers‐in‐law. In fact, grandmothers were eager to support mothers with child‐feeding and caring, creating a stronger bond between mothers and these elderly women. Utilizing indigenous foods for child feeding was observed as an opportunity for passing on traditional customs beneficial for child nutrition. Parity also had a strong influence on the belief in indigenous foods compared to modern feeding practices. New mothers were more receptive to the new foods, whereas those with older children first compared the new feeding practices to what they were accustomed to, questioning the new ways.

Based on historical community experiences, use of moringa was common among adults with diabetes and human immunodeficiency virus (HIV), as herbal tea, or spice.

‘I know it was introduced some years back when we were being taught about HIV’. [Mother 6; TIPS, FGD2]

Exploration of indigenous knowledge systems also exposed the knowledge gaps that existed in feeding practices, which were barriers to uptake of new foods. Such gaps included a lack of knowledge of the value of thick porridge for infants receiving solids; and the assumption that moringa powder was for adults with chronic health conditions, especially HIV. One mother was worried when she took moringa powder home and did not feed her child because she thought that the powder would affect the effectiveness of the antiretroviral drugs that her son was taking.

‘I was not sure what to do about moringa, so I just cooked the new recipes without it, does it not affect the way my child's ARV drugs work?’ [Mother 1; TIPS, FGD2]

### 3.4 Theme 4: Knowledge gaps on IYCF

Several IYCF knowledge gaps were identified during discussions with mothers. Most mothers did not know about biofortified or vitamin/mineral‐rich foods such as NUA45 sugar beans and provitamin A orange maize. Mothers who grew pro Vitamin A maize or NUA45 beans did not know of their benefits (Table 1).

‘Oh NUA45, we grow it in our rural area as a crop, but I had no idea it could benefit my child, I just used to like how it takes less time to cook’ [Mother 4; RF, FGD1]

Other caregivers reported a lack of knowledge on enriching children's meals using ground nutritious foods such as dried fish or edible insects. The importance of thick porridge was not commonly known among mothers and almost all mothers reported feeding their children thin porridge. This was due to their belief that infants have sensitive intestines and thick porridge or solid foods could damage their gut. Child feeding was not regarded as an important task and mothers would feed children whenever they found time in‐between their household chores, community or social activities. Time was not allocated for food preparation or for feeding the child (Table 1).

Almost no mothers fed solid protein‐rich foods between 6 and 11 months, as they were perceived as foods for older children, who could chew and swallow. These mainly included ASFs. Mothers preferred feeding young children *sadza* (thick, cooked maize‐meal) with meat soup rather than actual meat. Eggs were often not fed to young children due to cost and/or some health workers' advice (Table 2).

‘We just grew up knowing that children are not given foods such as meat and eggs. And ah eggs are now expensive… there is a doctor who once told me to stop giving my child eggs too early, he kept falling ill’. [Mother 9; RF, FGD1]

**Table 2:** Barriers and facilitators to optimal IYCF feeding behaviours that were identified.

**Time** ‘Chisi’*—*weekly day when work is forbidden^a^	**Cultural and religious beliefs** Basis for social interactions and platform for sensitization^a^ Beliefs as facilitators or barriers ‘I will not give my grandson anything with moringa, it's traditional medicine, that's against my religious beliefs’ [Paternal grandmother; RF_FGD 1]
**Experience** Confidence in foods grew with more practice at home^a^ ‘Cooking at home worked well for me, I grasped how to use them…’ [Mother Feedback_FGD3]	**Indigenous knowledge systems** Traditionally passed on feeding practices e.g. sour porridge, delayed introduction of solids ‘I started feeding mine solids when she was around 8 months old’ [Mother RF_FGD1]
**Gender dynamics** Women as implementers and silent decision makers^a^ Physically absent fathers	**Community ownership** Proactivity in coming up with long term ideas on continuity^a^ ‘If possible, we could do it as a project of growing beans, where you teach us what to do, even with eggs…’ [Mother 2 TIPS_FGD2]
**Family beliefs** Family Influence on attitudes and perception of foods Family support^a^ ‘my mother in law was excited and told me about other uses of these foods’ [Home visit 3_TIPS]	

Abbreviation: IYCF, infant and young child feeding.

^a^ Facilitators/Enablers.

## 4 DISCUSSION

We undertook this formative research to inform the design of an IYCF intervention utilized in the CHAIN child‐feeding trial study. The CHAIN trial study is a novel community‐based randomized trial designed to evaluate the effect of an enhanced infant feeding intervention (IYCF‐plus) on filling nutrient gaps for young children in rural Zimbabwe described elsewhere (Smith et al., 2022). The CHAIN trial leverages the agriculture‐nutrition nexus, embeds a context‐specific social behaviour change approach and utilizes a commercially available SQ‐LNS demonstrated to improve nutrient intake among young children in LMICs without replacing family foods (Arimond et al., 2015; Hemsworth et al., 2016; Thakwalakwa et al., 2015). In this study we tested the acceptability and feasibility of inclusion of powdered locally available foods, combined with behaviour change counseling, to address nutrient gaps among young children. Overall, we found that formulation of nutrient‐dense, high‐quality protein complementary meals using powdered locally available food supplements was feasible and highly accepted in this rural community. We also developed a comprehensive IYCF and behaviour change intervention with potential to improve complementary feeding practices among caregivers of children between 6 and 23 months living in under‐resourced communities.

Acceptability of the complementary food recipes was indicated by high sensory evaluation ratings. Higher sensory ratings among mothers who had more exposure to the food recipes could have been achieved by recipe improvements that were made as mothers became more comfortable with the ingredients during the TIPS period. In other African studies mothers were receptive to complementary foods incorporating familiar ingredients such as locally produced pulses (Anigo et al., 2010; Bisimwa et al., 2012; Fikiru et al., 2017). Use of moringa leaf powder, a traditional tree recognized in Zimbabwe and many LMICs for its functional benefits, enhanced acceptance of the new complementary food recipes. This is consistent with other studies in other LMICs (Boateng et al., 2018).

Mothers were receptive to the new food recipes as they could easily relate to local food ingredients, beans and eggs. The initial reaction of many mothers was that moringa is a herbal medicine, probably due to its wide use among people living with HIV during the pre‐ART era in Zimbabwe (Bepe et al., 2011; Chinsembu & Hedimbi, 2010; Monera & Maponga, 2012). However, after learning that moringa is a tree rich in micronutrients and protein it was readily accepted as a complementary food and regarded as safe. Moringa is widely accepted as a functional food rich in nutrients and has several health benefits in many African communities (Barichella et al., 2019; Boateng et al., 2018). Great interest was shown by grandmothers, grandfathers, fathers, and other family members in using moringa as a complementary food. This provides an opportunity for the integration of indigenous knowledge systems with recommended IYCF practices, resulting in increased support for mothers. Use of moringa, stimulated interest among grandmothers and other family members to become involved in child feeding, paving a way for increased support to mothers. Policymakers and IYCF programme practitioners could leverage this enthusiasm to improve the engagement of elderly women to support IYCF instead of regarding them as a barrier to uptake of optimal IYCF behaviours (Bernie, 2014; Fjeld et al., 2008; MoHCW, 2012; Nsiah‐Asamoah et al., 2020). Convergence of traditional foods and modern complementary feeding is a key theme that will be further investigated through a qualitative sub‐study in the CHAIN trial, to gain deeper understanding of how indigenous knowledge systems could be leveraged to improve IYCF.

Feeding young children ASFs has been reportedly poor in LMICs (Bwibo & Neumann, 2003; FNC, 2017; ZimVAC, 2020). Even in instances where small‐scale animal production was increased in LMICs, consumption of ASFs remained low, calling for innovative strategies to improve ASFs consumption (Broaddus‐Shea et al., 2020; Speedy, 2003). Increased egg production in rural areas is a low‐cost, sustainable approach. Developmental interventions incorporating egg production and consumption have demonstrated a positive impact on increasing protein quality in children's diets (Dumas et al., 2018; Iannotti et al., 2014, 2017). Currently, there are several barriers to feeding young children eggs; the shelf stability of eggs and the size and texture of an egg for a young infant. Egg powder could be a solution for regular feeding of eggs to young children. Egg powder has a long shelf‐life, which provides caregivers with a dependable and flexible source of eggs. Instead of relying upon day‐to‐day egg purchasing or production, egg powder can provide a consistent source that can be earmarked for the child. Additionally, one tablespoon of egg power (14 g) is equivalent to one egg, which can ensure that an adequate amount of egg is consumed in the small food quantities that young infants consume. Dehydrated egg powder has potential to be produced at home and in rural areas using solar and indigenous sun drying in low‐resourced communities of South Africa, India and other LMICs (Kenawi et al., 2015; Mnyandu et al., 2015). In the future, it may be possible to produce egg powder in rural communities either through farmer groups or individual households. This has potential to increase intake of high‐quality proteins among young children and improve household food security in rural areas. Thus, use of hydrolysed egg powder could be an effective solution to address this perennial deficit of ASFs in child diets (Agapova et al., 2018; Iannotti et al., 2017). This novel approach to enrich complementary foods with powders made from locally available nutrient‐dense foods could be potentially utilized to enrich nutrient content of complementary foods. This has become more relevant considering the World Health Organization's recent recommendation to use of nutrient‐dense family foods to prevent malnutrition and treat low‐risk moderate acute malnutrition (World Health Organization, 2023).

We observed that most mothers lived alone for long periods as their partners often migrate for work as small‐scale miners. In many sub‐Saharan African communities, this practice is common where men often travel away from home for long periods to mining areas, cattle camps, or fishing areas (Farnworth et al., 2015; Hanyani‐Mlambo et al., 2019). This enabled mothers to make decisions on child feeding without having to seek permission from male partners. Often IYCF projects are designed with a gender‐lens, utilizing programming, time, and resources to empower women and engage men—who are purported to be primary decision‐makers in child feeding. However, our findings offer an alternative view and call for further research to understand gender dynamics and the needs of women living in communities where overall decision‐making on agriculture and child feeding lies with them, whereas men often have an artificial sense of control or an assumed silent role. Qualitative research is planned to investigate the impact of gender dynamics on child feeding among women living in resource‐constrained areas such as rural Shurugwi and will be investigated in the CHAIN trial. Mothers who lived away from their mothers‐in‐law also demonstrated a sense of independence in making decisions on child‐feeding compared with mothers who lived with their mothers‐in‐law. Living with a mother‐in‐law took away the invisible emotional space mothers require to make decisions and explore behaviours and different routines in child feeding. Thus, the influence of the extended family and this emotional space for decision‐making and independence to explore new behaviours in child feeding require further investigation.

The novel participatory approach we employed in developing IYCF messages was effective in ensuring that they were easily understood by rural women. This approach is not common in developing IYCF behavior change communication (BCC) materials and overcomes a barrier to understanding and adoption of promoted behaviours (MoHCC, 2016; MoHCW, 2012; Girard et al., 2020). Mothers and CHWs emphasised the locally acceptable language, and types of easily understood images for BCC. This participatory IYCF message development approach showed potential for success in supporting behaviour change among women, which will be evaluated fully in the CHAIN trial.

Limitations relating to our study include potential high cost of producing and procuring powders from local foods for families in under‐resourced communities which could threaten the sustainable access to these nutrient‐dense powders. However, these foods are already produced in the community giving potential for a sustainable source of the products. Furthermore, TIPS demonstrate initial changes in the behaviour and not maintenance of the practices over a longer period.

## 5 CONCLUSION

In conclusion, suboptimal complementary feeding is a perennial challenge in rural communities of LMICs. We have established that a child feeding intervention package incorporating social and behaviour change with the provision of powdered egg, bean and moringa is acceptable in rural Zimbabwe. We have also identified several domains pertinent to adherence to the IYCF Plus intervention including food preparation time, experience with food ingredients, gender dynamics, cultural and religious beliefs, indigenous knowledge systems and community ownership of solutions (Table 2). Integrated complementary feeding interventions incorporating social and behaviour change, provision/promotion of specific nutrient‐dense complementary foods (SQ LNS and local foods), family support and agricultural production have potential for success in Zimbabwe and in other rural LMICs.

## AUTHOR CONTRIBUTIONS

Dexter T. Chagwena and Shamiso Fernando conducted the field research, analysed the data, and drafted the manuscript. Laura E. Smith, Jean H. Humphrey, Dexter T. Chagwena and Andrew J. Prendergast designed the research study and contributed to manuscript preparation. Shadreck Sithole, Naume V. Tavengwa, Chandiwana Nyachowe, Handrea Njovo, Kavita Datta, and Tim Brown provided essential input into study design and manuscript preparation. All authors contributed to writing and critical revision of the paper. All authors have read and approved the final manuscript.

## CONFLICT OF INTEREST STATEMENT

The authors declare no conflict of interest.

## Data Availability

The data that support the findings of this study are available from the corresponding author upon reasonable request.
